# NEAT1 regulates neuroglial cell mediating Aβ clearance via the epigenetic regulation of endocytosis-related genes expression

**DOI:** 10.1007/s00018-019-03074-9

**Published:** 2019-04-20

**Authors:** Ziqiang Wang, Yiwan Zhao, Naihan Xu, Shikuan Zhang, Songmao Wang, Yunhao Mao, Yuanchang Zhu, Bing Li, Yuyang Jiang, Ying Tan, Weidong Xie, Burton B. Yang, Yaou Zhang

**Affiliations:** 1 School of Life Sciences, Tsinghua University, Beijing, 100084 China; 2 Key Laboratory in Health Science and Technology, Division of Life Science, Graduate School at Shenzhen, Tsinghua University, Shenzhen, 518055 China; 3 State Key Laboratory of Oncology in South China, Collaborative Innovation Center of Cancer Medicine, Sun Yat-sen University Cancer Center, Guangzhou, 510060 China; 4 State Key Laboratory of Chemical Oncogenomics, Graduate School at Shenzhen, Tsinghua University, Shenzhen, 518055 China; 5 Open FIESTA Center, Tsinghua University, Shenzhen, 518055 China; 6 Department of Laboratory Medicine and Pathobiology, Sunnybrook Research Institute, University of Toronto, Toronto, Canada

**Keywords:** Alzheimer’s disease, Aβ, NEAT1, Histone modification, P300

## Abstract

The accumulation of intracellular β-amyloid peptide (Aβ) is important pathological characteristic of Alzheimer’s disease (AD). However, the exact underlying molecular mechanism remains to be elucidated. Here, we reported that Nuclear Paraspeckle Assembly Transcript 1 (NEAT1), a long n on-coding RNA, exhibits repressed expression in the early stage of AD and its down-regulation declines neuroglial cell mediating Aβ clearance via inhibiting expression of endocytosis-related genes. We find that NEAT1 is associated with P300/CBP complex and its inhibition affects H3K27 acetylation (H3K27Ac) and H3K27 crotonylation (H3K27Cro) located nearby to the transcription start site of many genes, including endocytosis-related genes. Interestingly, NEAT1 inhibition down-regulates H3K27Ac but up-regulates H3K27Cro through repression of acetyl-CoA generation. NEAT1 also mediates the binding between STAT3 and H3K27Ac but not H3K27Cro. Therefore, the decrease of H3K27Ac and/or the increase of H3K27Cro declines expression of multiple related genes. Collectively, this study first reveals the different roles of H3K27Ac and H3K27Cro in regulation of gene expression and provides the insight of the epigenetic regulatory mechanism of NEAT1 in gene expression and AD pathology.

**Electronic supplementary material:**

The online version of this article (10.1007/s00018-019-03074-9) contains supplementary material, which is available to authorized users.

## Introduction

Alzheimer’s disease (AD) is a chronic neurodegenerative disorder that deteriorates learning, memory and cognition [1]. This disease is characterized by the deposition of β-amyloid (Aβ) plaques and neurofibrillary tangles and neuronal and synaptic loss [2–4]. Aβ is generated via the cleavage of amyloid precursor protein (APP) by β and γ-secretases [5], and imbalanced Aβ production and degradation are thought to play vital roles in AD progression [6–9]. Therefore, stimulating amyloid clearance will help maximize the therapeutic reduction of neuropathology to prevent or arrest neurodegeneration and cognitive failure. An increasing number of studies has reported that neuroglial cells can remove the soluble, protofibrillar and fibrillar forms of Aβ via endocytosis and autophagy [10–13]. In addition, these cells participate in innate immunity and represent an important cell type that is responsible for AD progression [14].

Nuclear Paraspeckle Assembly Transcript 1 (NEAT1) is a long non-coding RNA (lncRNA) that exists in two isoforms: NEAT1v1 (3.7 kb in length) and NEAT1v2 (23 kb in length). Previous studies have reported that NEAT1 functions as an essential architectural component of paraspeckle nuclear bodies [15–18] with a gene expression regulatory function by retaining inverted Alu repeat-containing RNAs in the nucleus [19], physically binding to active chromatin sites [20], competitively sponging microRNAs [21–24] and associating with chromatin regulatory proteins, such as PRC1, PRC2, JARID1B, ESET and SUV39H1 [25]. Currently, NEAT1 has been shown to participate in the development of numerous diseases processes, such as corpus luteum formation [26], mammary gland development [27], cancers [28–30], viral infection [31–33] and autoimmune diseases [34, 35]. In addition, NEAT1 dysregulation has been examined in neurodegenerative diseases, such as Huntington’s disease [36] and multiple sclerosis [37]. However, no studies have explored the functions of NEAT1 in the progression of these neurodegenerative diseases.

In this study, we investigated the role of NEAT1 in AD progression and found that NEAT1 downregulation in AD inhibited the uptake of Aβ by regulating the transcriptional activities of endocytosis-related genes. Regarding the regulatory mechanism, NEAT1 mediates its autoacetylation of P300 and acyltransferase activity via an association with P300 and alters H3K27 acetylation and crotonylation of sites located near the transcription start sites (TSSs) of these gene promoters, which influences binding of the transcriptional factor STAT3 to these genes to initiate transcription. Taken together, our results reveal that NEAT1 is involved in regulation of Aβ endocytosis-related gene expression through epigenetic mechanisms. Thus, NEAT1 may provide a potential therapeutic target for AD intervention.

## Results

### NEAT1 regulates the Aβ clearance mediated by neuroglial cells

**Fig. 1:**
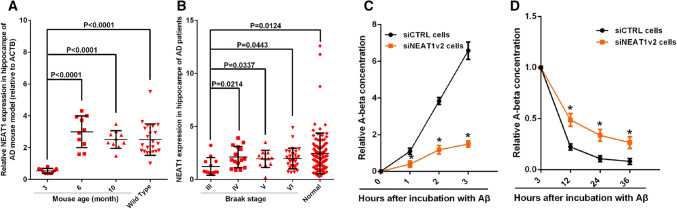
Downregulated NEAT1 in AD contributes to Aβ uptake and degradation. **a** NEAT1 analysis in the hippocampi of 3-month-old AD mice (*n* = 10), 6-month-old AD mice (*n* = 10), 10-month-old AD mice (*n* = 10) and 3-month-old wild-type mice (*n* = 22). *p* values were calculated using the nonparametric Kolmogorov–Smirnov test. **b** NEAT1 analysis in the hippocampi of different Braak stage AD patients and normal controls in the AD dataset (GSE48350). *p* values were calculated using the nonparametric Kolmogorov–Smirnov test. **c**, **d** After addition of Aβ (1–42) to the siNEAT1 and siCTRL cells for the indicated times, the relative Aβ (1–42) concentrations were analysed by ELISA in three independent experiments. The data are represented as the mean ± SD. *p < 0.001

To study amyloid deposition in AD, we utilized the APPswe/PS1dE9 double transgenic mouse model. We compared 3-, 6- and 10-month-old AD mice with normal mice and observed that expression of the long non-coding RNA NEAT1 was reduced in the hippocampi of the younger AD mice (3 months old) (Fig. 1a). Moreover, we used an AD dataset (GSE48350) from the National Center for Biotechnology Information (NCBI) database to analyse the NEAT1 expression levels in the AD hippocampi at different Braak stages and in normal hippocampi. As shown in Fig. 1b, NEAT1 expression was significantly decreased in the Braak III AD hippocampal tissue, which also exhibited neurofibrillary tangles [38]. The imbalance between Aβ production and clearance results in its aggregation into neuritic plaques [9]. Thus, we further investigated whether dysregulated NEAT1 in AD was involved in the uptake and clearance of Aβ. To achieve this aim, we investigated the effects of NEAT1 on Aβ endocytosis and degradation in the human astrocytic U251 cell line. First, we generated NEAT1-deficient U251 cells (siNEAT1v2) and negative control cells (siCTRL) using lentivirus-based NEAT1-targeting short hairpin RNA (shRNA) and control shRNA vectors, respectively (Fig S1A). β-Amyloid (1–42) (Aβ) was added to the siNEAT1v2 and siCTRL cell lines. Initially, neuroglial cells mainly function to endocytose Aβ. Therefore, we collected cells at 0, 1, 2 and 3 h after Aβ (1–42) addition and examined the cellular Aβ (1–42) concentrations with an enzyme-linked immunosorbent assay (ELISA). We found that Aβ uptake was markedly inhibited in the siNEAT1v2 cells compared with that of the siCTRL cells (Fig. 1c). To evaluate its effects on Aβ degradation, we harvested cells at 3, 12, 24 and 36 h after Aβ (1–42) addition and performed an ELISA. The Aβ (1–42) concentration relative to that at 3 h was calculated, and the decrease in the concentration range was used to characterize the degradation rate. As shown in Fig. 1d, NEAT1 knockdown inhibited Aβ degradation. To control for off-target effects, we introduced another siRNA [siNEAT1(v1 + v2)] that was used in our previous study [32] and found that these two siRNAs had similar effects on Aβ endocytosis and degradation (Fig S1B–D). Similar results were also obtained by flow cytometry (Fig S1E). Taken together, these results indicate that NEAT1 plays vital roles in neuroglial cell-mediated Aβ clearance.

### NEAT1 regulates endocytosis-related gene expression

**Fig. 2:**
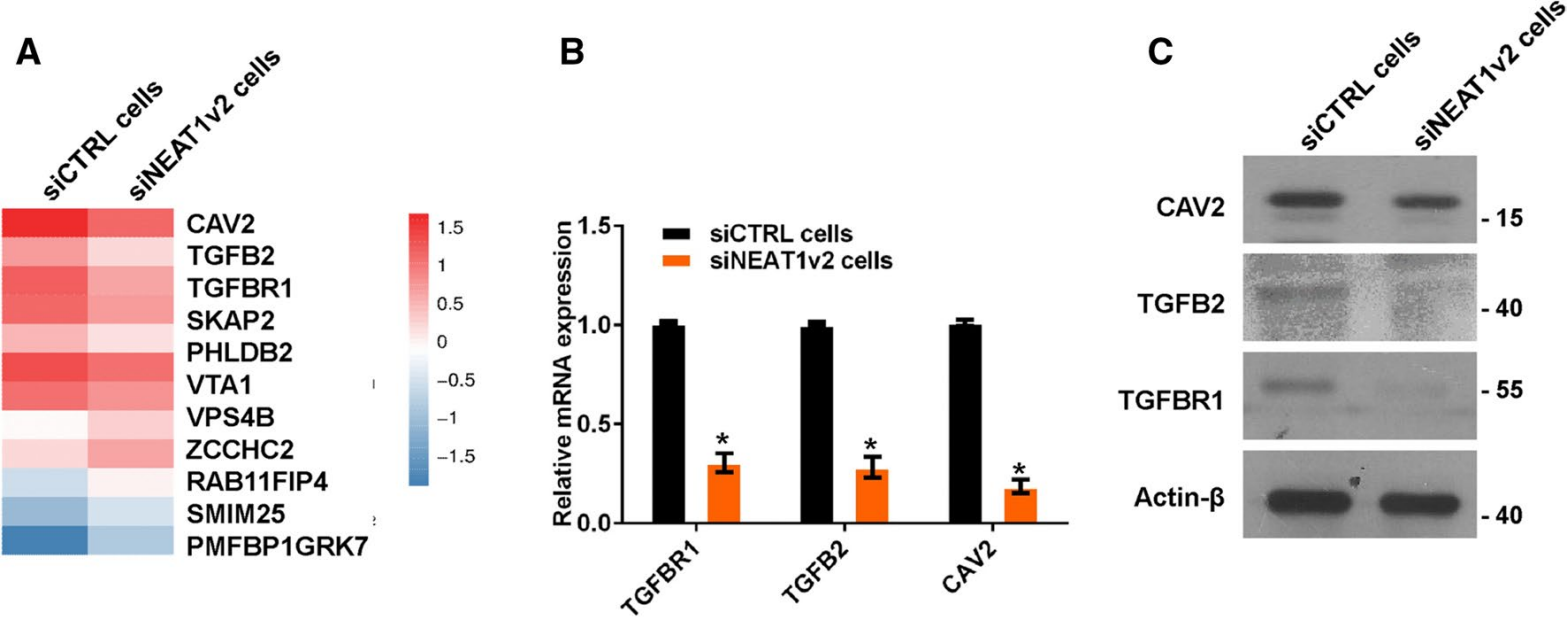
NEAT1 regulates endocytosis-related gene expression. **a** RNA-seq was performed in siNEAT1v2 and siCTRL cells, and endocytosis-related genes altered by NEAT1 were analysed. **b** The relative *CAV2*, *TGFB2* and *TGFBR1* mRNA levels in the siNEAT1v2 and siCTRL cells were analysed by qRT-PCR in three independent experiments. The data are represented as the mean ± SD. **c** The CAV2, TGFB2, TGFBR1 and β-actin protein levels in the siNEAT1v2 and siCTRL cells were measured by western blotting. *p < 0.001

To investigate the mechanism by which NEAT1 regulates Aβ uptake and degradation, we performed whole-genome RNA sequencing in siNEAT1v2 and siCTRL cells. The results revealed that the genes altered by NEAT1 (Fig S2A) participated in multiple pathways, including endocytosis (Fig S2B, red arrow), which is an important Aβ uptake method. We sorted these endocytosis-related genes based on fold changes and identified three genes encoding membrane proteins or membrane-binding proteins (Fig. 2a, CAV2, TGFB2 and TGFBR1). Furthermore, alterations of CAV2, TGFB2 and TGFBR1 mRNA and protein expression were confirmed by quantitative real-time PCR (qRT-PCR) and western blotting in siNEAT1v2 and siCTRL cells (Fig. 2b, c) and U251 cells transfected with the siNEAT1v2, siNEAT1(v1 + v2) or negative control siRNA (Fig S3). These results suggest that NEAT1 is involved in regulation of expression of the *CAV2*, *TGFB2* and *TGFBR1* endocytosis-related genes.

### CAV2, TGFB2 and TGFBR1 regulate Aβ endocytosis

**Fig. 3:**
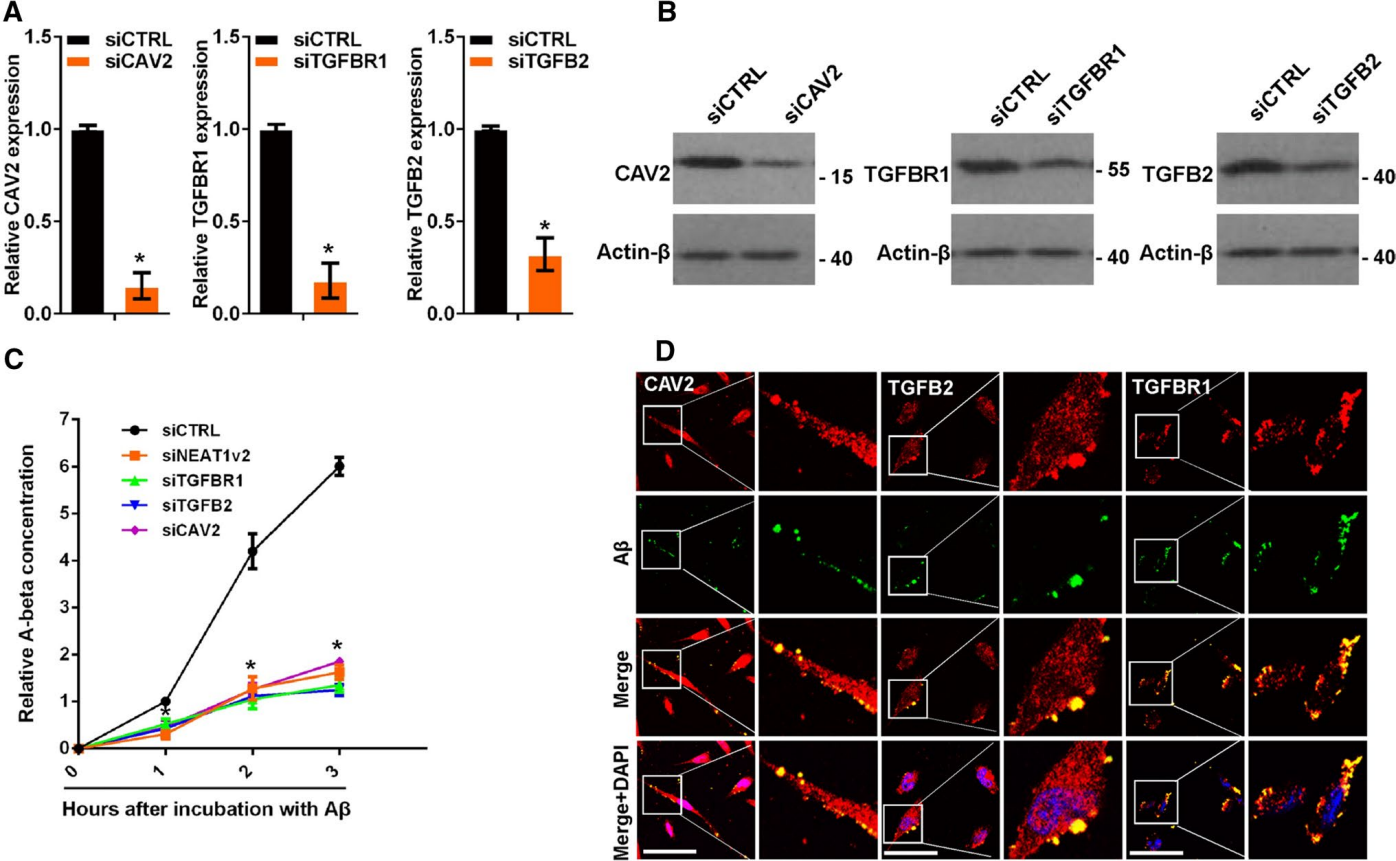
CAV2, TGFB2 and TGFBR1 were regulated by NEAT1-mediated Aβ endocytosis. **a** The relative *CAV2*, *TGFB2* or *TGFBR1* mRNA levels in U251 cells transfected with gene-targeting or negative control siRNAs were analysed by qRT-PCR in three independent experiments. The data are represented as the mean ± SD. **b** The CAV2, TGFB2 or TGFBR1 protein levels in U251 cells transfected with gene-targeting or negative control siRNAs were measured by western blotting. **c** After addition of Aβ (1–42) to U251 cells transfected with the gene-targeting or negative control siRNAs for the indicated times, the relative Aβ (1–42) concentration was measured by ELISA in three independent experiments. The data are represented as the mean ± SD. **d** After the addition of HiLyte Fluor™ 488-labelled Aβ (1–42) for 1 h, U251 cells were immunostained with CAV2, TGFB2 or TGFBR1, and the relationship between Aβ (green) and CAV2 (red), TGFB2 (red) or TGFBR1 (red) was visualized by confocal microscopy. Scale bars 25 μm. *p < 0.001

To investigate the effects of CAV2, TGFB2 and TGFBR1 on Aβ endocytosis, we transfected U251 cells with siRNA targeting CAV2, TGFB2, or TGFBR1 or a negative control (Fig. 3a, b). Aβ (1–42) was added to the cells for 0, 1, 2 and 3 h, and the cellular Aβ (1–42) concentrations were examined by ELISA. CAV2, TGFB2 and TGFBR1 knockdown markedly inhibited Aβ uptake (Fig. 3c). CAV2 and TGFBR1 are membrane proteins, and TGFB2 is a membrane-binding protein. Therefore, we examined whether these proteins could co-localize with Aβ to aid in its endocytosis. We performed immunofluorescence experiments to detect potential interactions among CAV2, TGFB2, TGFBR1 and Aβ. As expected, CAV2, TGFB2 and TGFBR1 co-localized with Aβ (Fig. 3d). Taken together, these results demonstrated that CAV2, TGFB2 and TGFBR1 regulated Aβ endocytosis.

### NEAT1 regulates the transcriptional activity of *CAV2*, *TGFB2* and *TGFBR1*

**Fig. 4:**
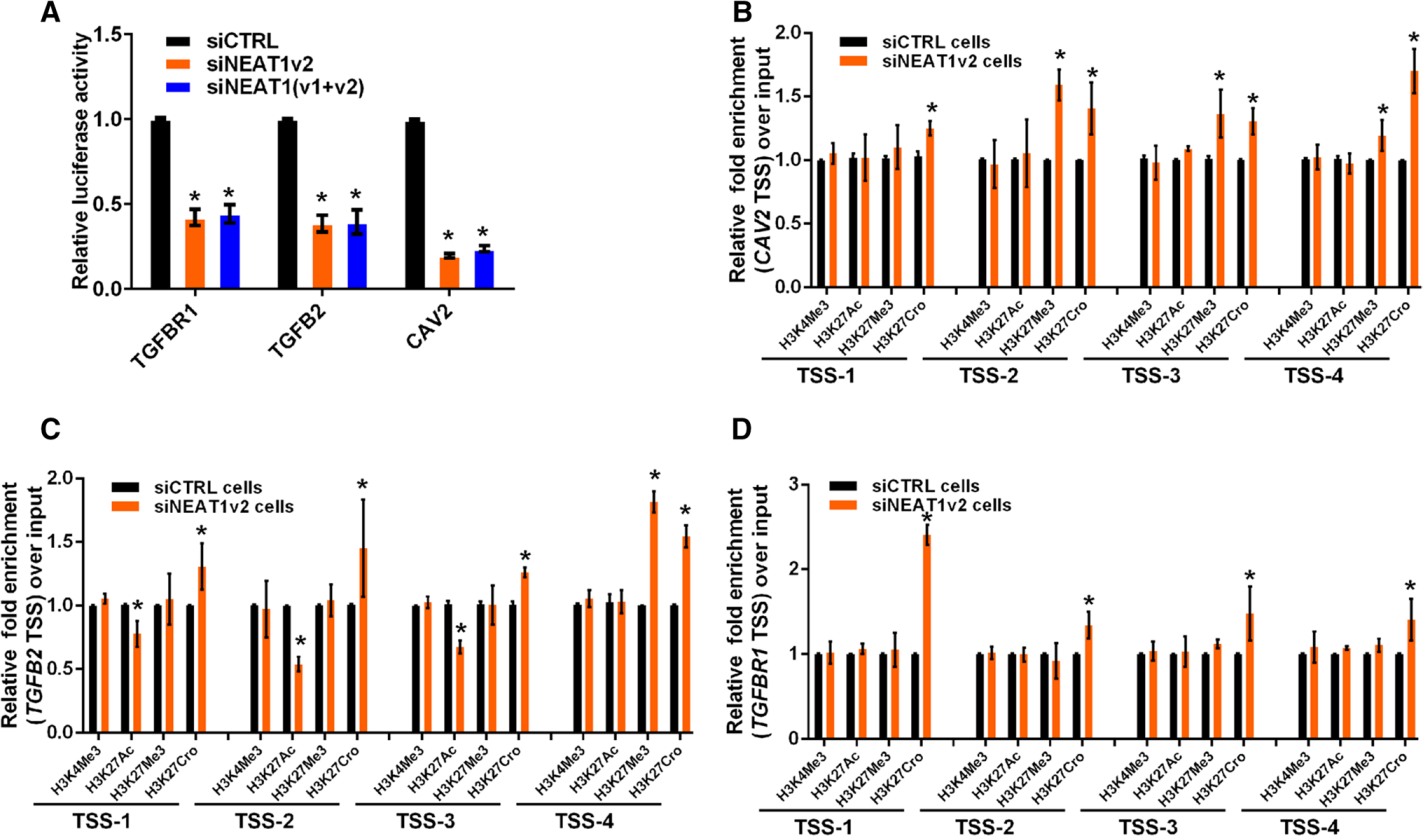
NEAT1 regulates the transcriptional activities of endocytosis-related genes through regulating histone modifications. **a** After co-transfection with siNEAT1v2, siNEAT1(v1 + v2) or negative control siRNAs and the pGL3 enhancer plasmid containing the CAV2, TGFB2 or TGFBR1 promoter for 36 h, the relative transcriptional activities of these promoters were determined with a luciferase assay in three independent experiments. The data are represented as the mean ± SD. The siNEAT1 and siCTRL cells were collected for ChIP assays to analyse the relative fold enrichment of the *CAV2* (**b**), *TGFB2* (**c**) and *TGFBR1* promoters (**d**) using anti-H3K4Me3, H3K27Ac, H3K27Me3 or H3K27Cro antibodies. The data points represent mean values determined from three independent experiments. The data are presented as the mean ± SD. *p < 0.001

Recently, we and other researchers reported that NEAT1 functioned as a transcriptional regulator to mediate gene expression [32, 39]. To elucidate the mechanism by which NEAT1 regulated *CAV2*, *TGFB2* and *TGFBR1* gene expression, we performed luciferase assays to examine whether NEAT1 directly regulated the transcriptional activities of these genes. Three constructs (luciferase reporters containing *CAV2*, *TGFB2* or *TGFBR1* promoter fragments) were used in the luciferase activity assay. Our results demonstrated that NEAT1 knockdown inhibited transcriptional activity at the *CAV2*, *TGFB2* and *TGFBR1* promoters in U251 cells (Fig. 4a). Additionally, we examined the histone modification status at these gene promoters in siNEAT1v2 and siCTRL cells. We performed chromatin immunoprecipitation (ChIP) experiments using antibodies against tri-methylated histone H3 at lysine 4 (H3K4Me3), acetylated histone H3 at lysine 27 (H3K27Ac), tri-methylated histone H3 at lysine 27 (H3K27Me3) and crotonylated histone H3 at lysine 27 (H3K27Cro). The presence of H3K4Me3 and H3K27Ac at TSS serves as a marker for actively transcribed genes, whereas H3K27Me3 in TSSs is associated with gene repression [40]. H3K27Cro is a newly identified modification with unknown functions [41]. To identify these modified histone H3-binding sites within the *CAV2*, *TGFB2* and *TGFBR1* promoter sequences, we designed sets of primer pairs (Table S1) that recognized the TSS regions of the *CAV2*, *TGFB2* and *TGFBR1* genes (Fig S4). The ChIP assay demonstrated that knocking down NEAT1 increased the enrichment of H3K27Cro at the *CAV2*, *TGFB2* and *TGFBR1* promoters and decreased the enrichment of H3K27Ac at the *CAV2* and *TGFBR1* promoters (Fig. 4b–d). Specifically, decreased H3K27Ac and increased H3K27Cro were found near TSSs of the *TGFBR1* promoter (Fig. 4b). Taken together, our results demonstrated that NEAT1 regulated *CAV2*, *TGFB2* and *TGFBR1* transcriptional activity by altering the histone modification statuses of these gene promoters.

### Crotonyl-CoA regulates endocytosis-related gene expression

**Fig. 5:**
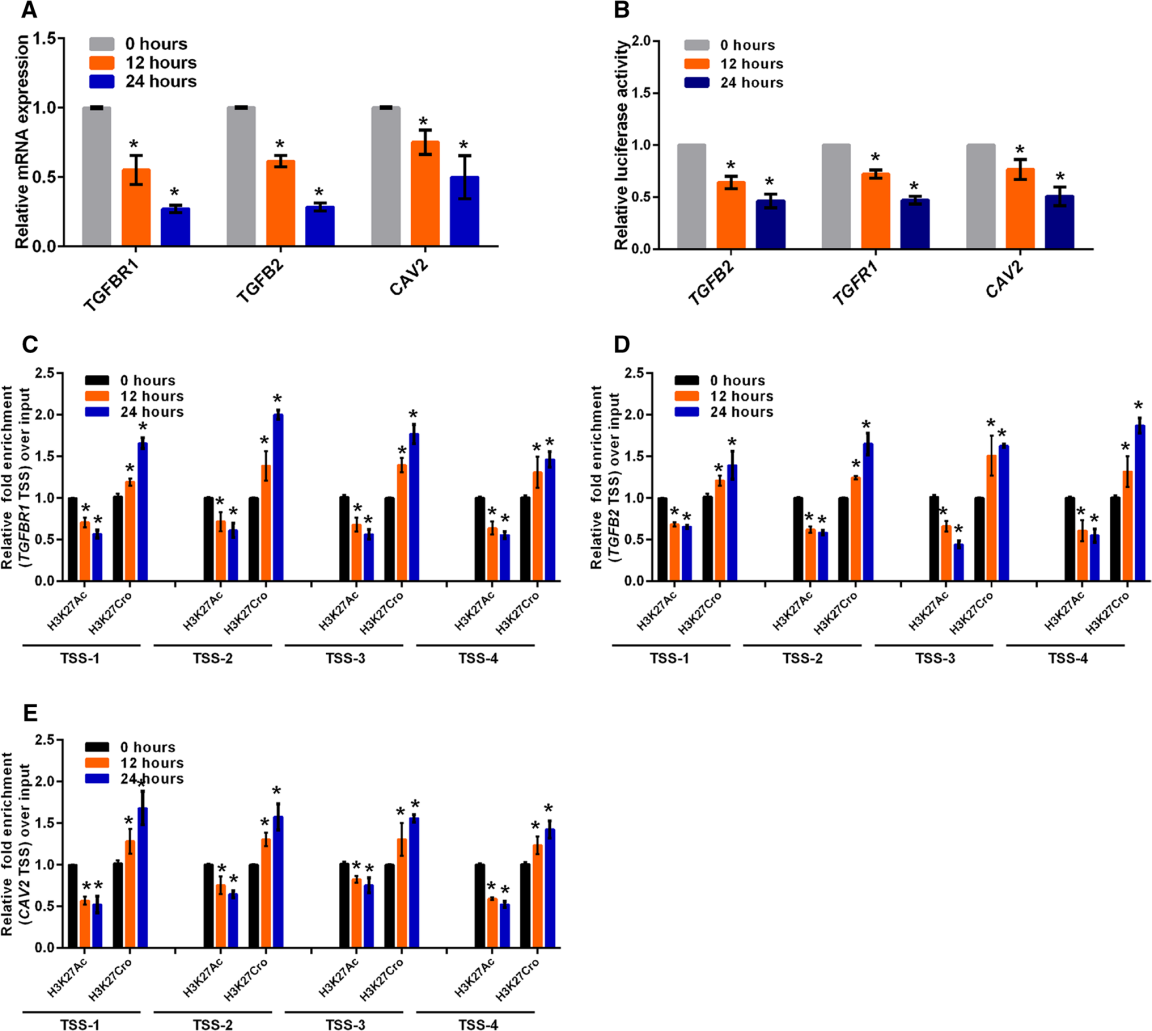
Crotonyl-CoA regulates endocytosis-related gene expression. **a** The relative CAV2, TGFB2 and TGFBR mRNA levels were analysed by qRT-PCR in U251 cells incubated with 80 µm crotonyl-CoA for the indicated times. The data points represent the mean values determined from three independent experiments. The data are presented as the mean ± SD. **b** After incubation with 80 µm crotonyl-CoA for the indicated times, U251 cells were transfected with the pGL3 enhancer plasmid containing the CAV2, TGFB2 or TGFBR1 promoter for 36 h. The relative transcriptional activities of these promoters were determined with a luciferase assay in three independent experiments. The data are represented as the mean ± SD. U251 cells incubated with 80 µm crotonyl-CoA for the indicated times were collected for ChIP assays to analyse the relative fold enrichment of the *TGFBR1* (**c**), *TGFB2* (**d**), or *CAV2* promoter (**e**) using anti-H3K27Ac or H3K27Cro antibodies. The data points represent the mean values determined from three independent experiments. The data are presented as the mean ± SD. *p < 0.001

Given that histone modification via crotonylation is mediated by cellular crotonyl-CoA and potentially affects gene expression [42], we added different crotonyl-CoA concentrations to the culture media to assess the effects of the changes in histone crotonylation on targeted gene expression. TGFBR1 expression was decreased 24 h after incubation with different crotonyl-CoA concentrations (5–80 µm), increased 12 h after incubation with 10 µm crotonyl-CoA and gradually declined thereafter after incubation with the higher crotonyl-CoA concentrations (Fig S5A). To investigate the effect of high crotonyl-CoA concentrations on cell cytotoxicity in vitro, the Cell Counting Kit-8 (CCK8) assay was performed in U251 cells incubated with 80 µm crotonyl-CoA. The results revealed no significant effect on the cell viability or proliferative capacity (Fig S5B). Furthermore, we examined their expression levels in U251 cells 24 h after incubation with 80 µm crotonyl-CoA. The addition of exogenous crotonyl-CoA significantly inhibited the expression of endocytosis-related genes in these cells (Fig. 5a).

To assess whether exogenous crotonyl-CoA inhibited the transcriptional activity of these genes via increasing the level of crotonylation modification at the gene promoters, we conducted luciferase assays and ChIP experiments. We found that exogenous crotonyl-CoA inhibited the transcriptional activity of these genes (Fig. 5b), increased H3K27 crotonylation and attenuated H3K27 acetylation at the gene promoters (Fig. 5c–e). These results showed that a higher intracellular Crotonyl-CoA concentration inhibited the transcription activities and expression of endocytosis-related genes through regulation of H3K27 crotonylation and acetylation, verifying that alteration of the H3K27 crotonylation modification at these gene promoters mediated by NEAT1 regulated related gene expression.

### NEAT1 regulates the acetylation and crotonylation of multiple proteins

**Fig. 6:**
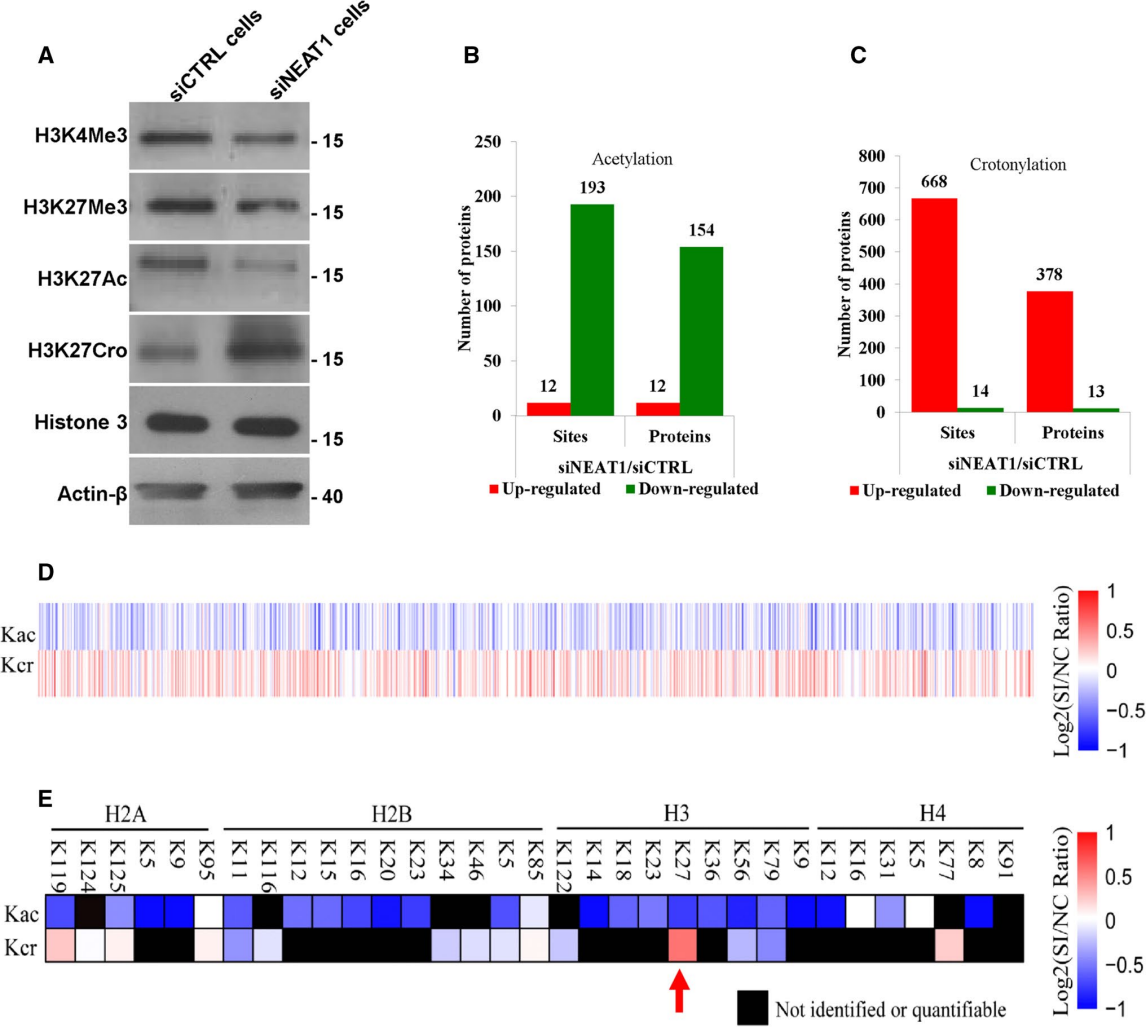
NEAT1 regulates the acetylation and crotonylation of histone and non-histone proteins. **a** The H3K4 H3K4Me3, H3K27Me3, H3K27Ac, H3K27Cro, Histone 3 and β-actin levels in the siNEAT1v2 and siCTRL cells were measured by western blotting. A modification-specific (lysine acetylation and crotonylation) proteomics analysis was performed in the siNEAT1v2 and siCTRL cells. The numbers of acetylation (**b**)- and crotonylation (**c**)-modified sites and proteins regulated by NEAT1 were analysed, and then the acetylation and crotonylation of non-histone (**d**) and histone proteins (**e**) regulated by NEAT1 were sorted out

To further study the molecular mechanism by which NEAT1 regulated histone crotonylation and acetylation at the TSSs of the target genes, we analysed the effect of NEAT1 on the global levels of various histone modifications. The results revealed a decrease in H3K4Me3, H3K27Me3 and H3K27Ac and an increase in H3K27Cro in the NEAT1 knockdown cells (Fig. 6a) and the U251 cells transfected with siNEAT1v2 and siNEAT1(v1 + v2) (Fig S6). Given that histone acetylation and histone crotonylation require the same acyltransferase (i.e., P300) and are involved in epigenetic regulation of gene expression [43, 44], we focused on the roles of NEAT1 in regulating these two histone modifications. To investigate whether NEAT1 regulated the acetylation and crotonylation of other histone lysine sites or non-histone proteins, we conducted a modification-specific proteomics study. NEAT1 regulated the acetylation (Fig. 6b) and crotonylation levels (Fig. 6c) of a variety of proteins, and an opposite trend was noted for multiple loci (Fig. 6d). Specifically, knockdown of NEAT1 increased H3K27Cro and decreased H3K27Ac (Fig. 6e, red arrow), which was consistent with the results of the western blotting experiment. These results suggest that NEAT1 is a potentially important epigenetic regulatory factor in gene expression.

### NEAT1 influences acetyl-CoA generation and autoacetylation of P300

**Fig. 7:**
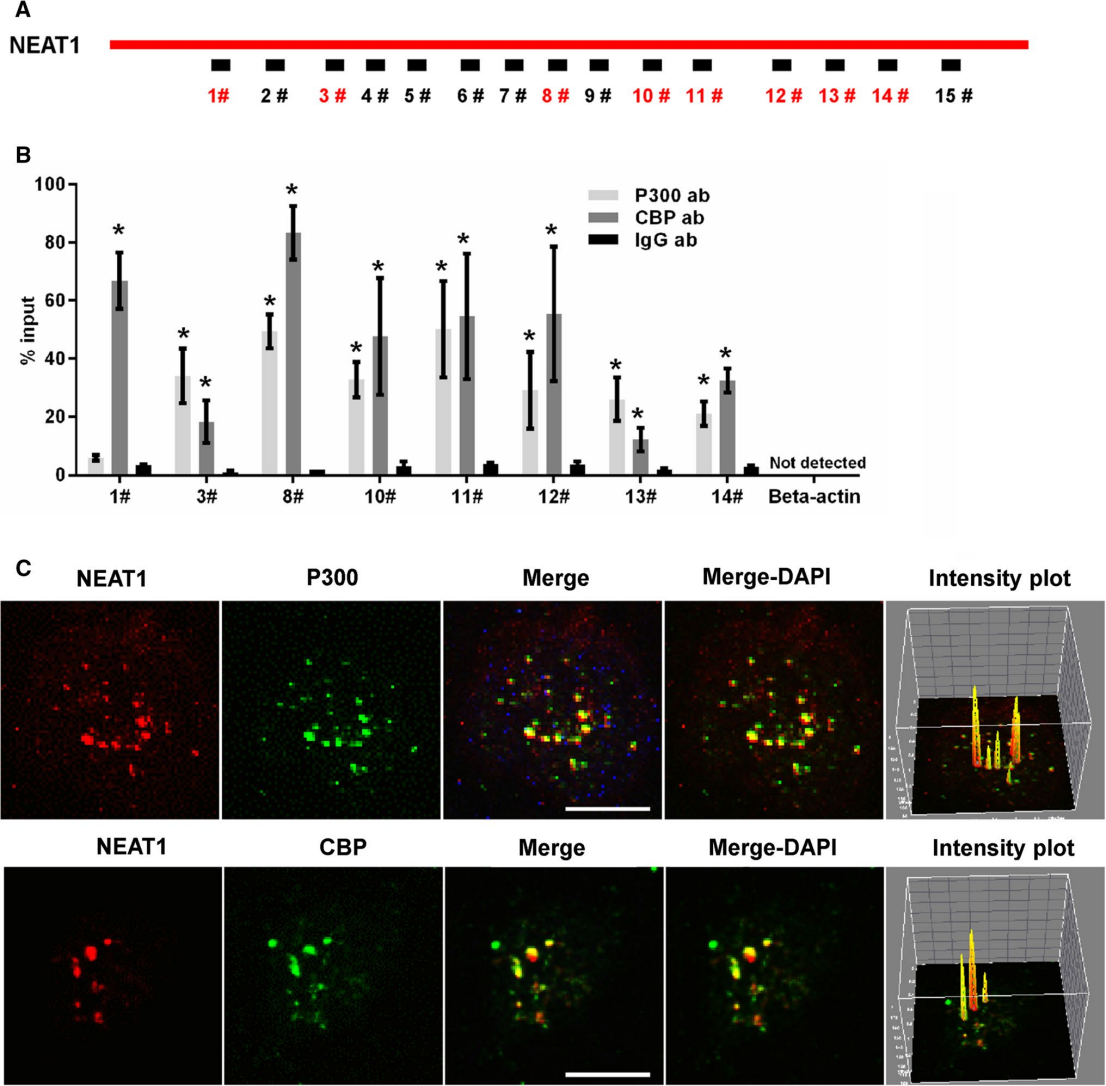
NEAT1 associated with P300/CBP. **a** Schematic of potential P300/CBP binding sites in the NEAT1 sequence. The box represents the primer-amplified regions, and the red boxes show the potential binding sites. **b** The U251 cells lysates were harvested and subjected to a RIP assay. QRT-PCR was performed to detect the retrieval of NEAT1 and β-actin by the anti-P300, anti-CBP or anti-IgG antibodies over the input level. The data points represent the mean values determined from three independent experiments. The data are presented as the mean ± SD. **c** The U251 cells were fixed and incubated with a NEAT1 probe (red) and then anti-P300 (green) or anti-CBP antibodies (green) before the confocal analysis. The intensity plots for the red and green channels were analysed with the ImageJ software. Scale bars 10 μm. *p < 0.001

To explore the mechanism by which NEAT1 regulated the H3K27Ac and H3K27Cro levels, we examined the influence of NEAT1 on the level of acetyl-CoA, which is the acyl-donor of protein acetylation, and found that U251 cells transiently transfected with siNEAT1v2 and siNEAT1(v1 + v2) for 72, 96 and 120 h exhibited lower acetyl-CoA levels (Fig S7A). In addition, we found that downregulation of NEAT1 expression could inhibit the autogenous acetylation levels of histone acetyltransferase P300 (P300-Ac) (Fig S7B). Given that P300 autoacetylation is related to P300 acetyltransferase activity [43], NEAT1 potentially influences the acetylation and crotonylation of histones by regulating the P300 autoacetylation process. To further explore how NEAT1 influenced P300 autoacetylation, we performed an RNA immunoprecipitation (RIP) assay. U251 cell lysates were harvested and subjected to an immunoprecipitation assay with the P300 antibody, CBP antibody or IgG antibody followed by qRT-PCR using primers that recognized NEAT1 fragments (Fig. 7a). The antibody to P300 pulled down fragments 3, 8, 10, 11, 12, 13 and 14 of NEAT1, and the antibody to CBP pulled down fragments 1, 3, 8, 10, 11, 12, 13 and 14 of NEAT1 (Fig. 7b). Next, we performed RNA fluorescence in situ hybridization (RNA-FISH) and immunofluorescence assay experiments. As shown in Fig. 7c, NEAT1 largely colocalized with P300 and CBP. Additionally, pixel intensity plots were generated for each merged channel (Fig. 7c, right panels). These data are the first to demonstrate that NEAT1 recognizes and colocalizes with the histone acetyltransferase complex P300 and CBP and indicate that NEAT1 affects its autoacetylation and acyl-transferase activities by direct interaction with P300.

### NEAT1 influences the association of transcriptional factor STAT3 with targeted genes

**Fig. 8:**
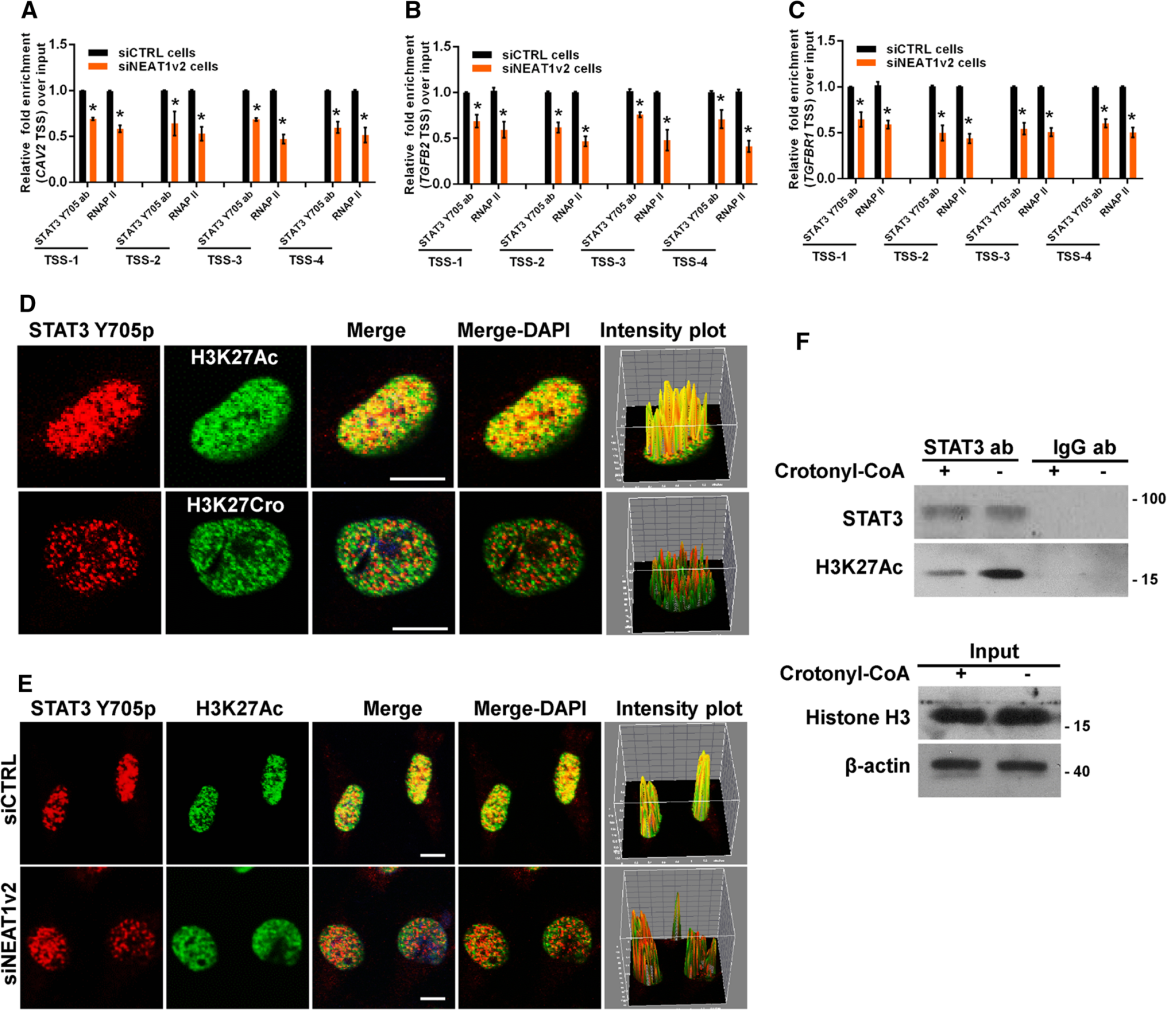
NEAT1 influences STAT3 binding to endocytosis-related genes. The siNEAT1 and siCTRL cells were collected for ChIP assays to analyse the relative fold enrichment of the *CAV2* (**a**), *TGFB2* (**b**), or *TGFBR1* promoter (**c**) with anti-STAT3 or RNAP II antibodies. The data points represent the mean values determined from three independent experiments. The data are presented as the mean ± SD. **d** The U251 cells were fixed and incubated with anti-STAT3 (red), anti-H3K27Ac (green) or anti-H3K27Cro antibodies (green) before the confocal analysis. The intensity plots for the red and green channels were analysed with the ImageJ software. Scale bars 10 μm. **e** After transfection with the siNEAT1v2 or negative control siRNA, the U251 cells were fixed and incubated with anti-STAT3 (red) or anti-H3K27Ac antibodies (green) before the confocal analysis. The intensity plots for the red and green channels were analysed with the ImageJ software. Scale bars 10 μm. **f** After 24 h of incubation with 80 µm crotonyl-CoA or the mock control, the U251 cell lysates were harvested and subjected to an immunoprecipitation assay with anti-STAT3 or anti-IgG antibodies. The retrieval of STAT3, H3K27Ac, H3K27Cro+ and Histone H3 by endogenous STAT3 and IgG was measured by western blotting. *p < 0.001

Previously, we reported that NEAT1 scaffolded transcriptional factor STAT3 to binding to NEAT1-targeted genes and regulation of the expression of these genes [32]. Thus, we hypothesized that STAT3 was involved in the NEAT1-mediated effects on cell endocytosis-related gene expression. As expected, the general consensus STAT3-binding motif [45, 46] was identified at the TSSs of these genes (Fig S8), and ChIP assays revealed that enrichment of STAT3-associated gene fragments was significantly reduced after NEAT1 depletion (Fig. 8a–c). However, the immunofluorescence assay demonstrated that STAT3 only co-localized with H3K27Ac and not H3K27Cro (Fig. 8d) and that inhibition of endogenous NEAT1 with siNEAT1v2 could disrupt this association between STAT3 and H3K27Ac (Fig. 8e). Furthermore, the immunoprecipitation assay results also demonstrated that STAT3 could not pull down H3K27Cro and that an increase in the cellular H3K27Cro level by addition of exogenous crotonyl-CoA to the cell culture media resulted in a significant reduction in the interaction of STAT3 with H3K27Ac (Fig. 8f). These findings indicated that the inhibitory effect of H3K27Cro on gene expression was related to the competitive decrease of the interaction of H3K27Ac with STAT3 caused by the increase in H3K27Cro, which could not bind STAT3. These data suggest that NEAT1 is required for the recruitment of STAT3 to H3K27Ac to upregulate related gene expression and that downregulated NEAT1 disrupts this association by upregulation of H3K27Cro, which cannot interact with STAT3.

## Discussion

AD is a neurodegenerative disease that exhibits the histopathological hallmark of the accumulation of Aβ-containing neuritic plaques. During AD progression, neuroglial cells take up and eliminate Aβ through cellular endocytosis [47]. In this study, we found that NEAT1, which is a long non-coding RNA that is important for nuclear paraspeckle formation, participated in neuroglial cell-mediated Aβ endocytosis by altering histone modifications near the TSSs of endocytosis-related genes.

In the early stage of AD, NEAT1 exhibits reduced hippocampal expression. We found that depletion of NEAT1 resulted in attenuation of Aβ uptake and degradation during the onset and at later stages of endocytosis, respectively. We and other researchers previously reported that NEAT1 regulated the expression of multiple genes by modulating their transcriptional activities [20, 32, 39]. Thus, we assessed the expression of genes altered by NEAT1 knockdown in whole-genomic RNA and identified several genes related to cellular endocytosis that exhibited altered expression levels. The internalization of oligomeric or fibrillary Aβ requires a number of receptors for endocytosis [48]. Thus, we chose three genes encoding membrane or membrane-binding proteins (i.e., *CAV2*, *TGFB2* and *TGFBR1*) for further study. To date, numerous receptors on the surface of neuroglial cells have been reported to be involved in Aβ endocytosis [49–57]. Here, we identified three new proteins regulated by NEAT1 that co-localized with and mediated Aβ endocytosis.

As the architecture of paraspeckles, NEAT1 regulates gene transcription via nuclear retention of mRNAs [58], sequestration of transcription factors from the promoters of target genes [59] or directly binding to the TSS regions of these genes [32]. To determine the regulatory mechanism of NEAT1 in targeted gene expression, we investigated the effects of NEAT1 on the histone H3 modification status (H3K4Me3, H3K27Me3, H3K27Ac and H3K27Cro) of the TSSs of these genes and found that NEAT1 altered at least one type of histone H3 modification on these genes. In addition, our results indicate that H3K27Cro, which is a newly identified reversible modification, may correlate with transcriptional repression.

Although histone acetylation and crotonylation are dynamically regulated by the acyltransferase P300, how these two modifications are regulated to maintain balance remains unclear. This study reveals that the long non-coding RNA NEAT1 is a key molecule that regulates these two modifications. In this investigation, we found that NEAT1 interacted with the acetyltransferase complex P300 and CBP and that silencing NEAT1 expression not only downregulated H3K27Ac but also upregulated the H3K27Cro level, which might be caused by the NEAT1-mediated decrease of acetyl-CoA generation. Acetyl-CoA and crotonyl-CoA are substrates of H3K27Ac and H3K27Cro, respectively. A competitive relationship was reported between acetyl-CoA and crotonyl-CoA for binding with H3K27 [42]. Ultimately, the decrease in acetyl-CoA may cause the reduction of H3K27Ac/H3K27Cro. H3K27Cro does not interact with STAT3, whereas H3K27Ac can bind STAT3 with the aid of NEAT1. The decrease in H3K27Ac and/or the increase in H3K27Cro ultimately downregulate the expression of their target genes. Although histone crotonylation was identified as early as 2011 [41], its role in the regulation of gene expression has not been completely characterized [43]. Some researchers have hypothesized that crotonylation is similar to histone acetylation and plays a role in promoting gene transcription through its association with active chromatin [42]. In the current study, we report that H3K27Ac and H3K27Cro play different roles in the regulation of gene expression.

Taken together, our findings reveal the regulatory mechanism of NEAT1 in β-amyloid deposition, which has significant implications for the aetiology of AD and may provide a new intervention target for preventing or delaying the occurrence of AD.

## Materials and methods

### Mice

Wild-type and APPswe/PS1dE9 double transgenic mice (an AD mouse model) were purchased from Jackson Laboratory (Bar Harbor, ME, USA) and housed in the SPF animal facility of Tsinghua University in individually ventilated cages. To detect NEAT1 expression in hippocampal tissue, the AD and WT mice were sacrificed by decapitation. Hippocampal tissue was dissociated immediately on ice and stored in liquid nitrogen until needed. All mice used in this study were handled according to the protocols approved by the Animal Welfare and Ethics Committee of Tsinghua University.

### Dataset

The Alzheimer’s Disease Dataset (GSE48350) was downloaded from the Gene Expression Omnibus (GEO, https://www.ncbi.nlm.nih.gov/gds/) database. NEAT1 expression in the hippocampi of AD patients at different stages and normal controls was analysed with the nonparametric Kolmogorov–Smirnov test.

### Generation of the stable cell lines and cell culture

The lentivirus-based NEAT1-targeting shRNA and control shRNA vectors were purchased from GenePharma Co. (Shanghai, China). U251 cells were transiently transfected with these vectors, followed by puromycin selection at a final concentration of 10 μg/ml to generate two stable monoclonal cell lines (siNEAT1v2 cells and siCTRL cells, respectively). The U251, siNEAT1v2 and siCTRL cells were grown in Dulbecco’s modified Eagle’s medium (DMEM, Gibco/Invitrogen Ltd., 12800-017) containing 10% foetal bovine serum (PAA, A15-101) and 10 U/ml of penicillin–streptomycin (Gibco/Invitrogen Ltd., 15140-122) in a 5% CO_2_-humidified incubator at 37 °C.

### Aβ (1–42) peptide quantitation by ELISA

To evaluate the roles of NEAT1, CAV2, TGFB2 and TGFBR1 in Aβ (1–42) uptake, U251 cells transfected with the gene-targeting or negative control siRNAs were inoculated with Aβ (1–42) (MERCK, AG970-1MG). After 0, 1, 2 and 3 h, the cells were washed and collected and the Aβ (1–42) levels were measured with the Amyloid Beta 42 Human ELISA Kit (Thermo Scientific, KHB3441) according to the manufacturer’s protocol. The data were normalized to the Aβ (1–42) concentration in the siCTRL cells incubated with Aβ (1–42) for 1 h. To evaluate the role of NEAT1 in Aβ (1–42) degradation, U251 cells transfected with NEAT1-targeting or negative control siRNAs were inoculated with Aβ (1–42) (MERCK, AG970-1MG). Three hours later, the cells were washed and re-suspended. After 9, 21 and 33 h, the cells were washed and collected, and the Aβ (1–42) levels were measured with the Amyloid Beta 42 Human ELISA Kit (Thermo Scientific, KHB3441) according to the manufacturer’s protocol. The data were normalized to the Aβ (1–42) concentration in the cells incubated with Aβ (1–42) for 3 h.

### Cell transfection, RNA isolation, reverse transcription and qPCR

All synthetic siRNAs and the negative control (NC) were purchased from Shanghai GenePharma Co., Ltd. All siRNAs were transfected with Lipofectamine™ 2000 (Invitrogen, 11668-019) according to the manufacturer’s protocol. For plasmid transfection, the cells were transiently transfected with Lipofectamine™ 3000 (Invitrogen, 1656200). Total RNA was isolated using RNAiso Plus (Takara, D9108B) according to the manufacturer’s protocol. Real-time qRT-PCR was performed using the ReverTra Ace^®^ qPCR RT Master Mix with gDNA remover (TOYOBO, FSQ-301) and the SYBR Green PCR Master Mix (TOYOBO, QPK-201). All mRNA levels were measured and normalized to β-actin. The primers used are listed in Table S1.

### RNA sequencing

Total RNA was isolated using RNAiso Plus (Takara, D9108B) according to the manufacturer’s protocol, and mRNA was purified from the total RNA using poly-T oligo-attached magnetic beads. RNA degradation and contamination were monitored on 1% agarose gels. The RNA purity was checked using the NanoPhotometer^®^ spectrophotometer (IMPLEN, CA, USA). The RNA integrity was assessed using the RNA Nano 6000 Assay Kit for the Agilent Bioanalyzer 2100 system (Agilent Technologies, CA, USA). Libraries were sequenced on the Illumina HiSeq 2500 platform. Differentially expressed genes were identified using the DESeq package with standard settings. Genes with a *p* adjust < 0.05 were considered differentially expressed. The differentially expressed mRNAs were used for KEGG enrichment analysis with the KOBAS software.

### Western blotting

The cells were lysed in ice-cold whole cell extract buffer B (50 mM TRIS–HCl, pH 8.0, 4 M urea and 1% Triton X-100) supplemented with a complete protease inhibitor mixture. The cell extracts were resolved by SDS-PAGE and analysed by western blotting. Protein bands were visualized using ECL Blotting Detection Reagents. The antibodies used for western blotting include CAV2 (Abcam, ab133484), TGFB2 (Abcam, ab36495), TGFBR1 (Abcam, ab31013) and β-actin antibodies (Proteintech, 60008-1-Ig).

### Immunofluorescence microscopy

To investigate the interaction between Aβ and CAV2, TGFB2 or TGFBR1, U251 cells were inoculated with β-amyloid (1–42), HiLyte Fluor™ 488-labelled (ANASPEC, AS-60479-01). After 1 h, the cells were washed, fixed and incubated with CAV2 (Abcam, ab133484), TGFB2 (Abcam, ab36495) or TGFBR1 antibodies (Abcam, ab31013). The cells were washed, counterstained with DAPI and observed under an Olympus FV1000 confocal laser microscope. To study the interaction between NEAT1 and P300/CBP, U251 cells were incubated with the NEAT1 probe overnight at 37 °C and then the anti-P300 (Abcam, ab59240) or anti-CBP antibodies (Abcam, ab50702) for 1.5 h at room temperature. After the cells were washed and incubated with the secondary antibody, they were counterstained with DAPI and mounted for observation. Cell images were obtained with an Olympus FV1000 confocal microscope. To study the role of NEAT1 in the interaction between STAT3 and H3K27Ac, U251 cells were transfected with a NEAT1 or negative control siRNA for 36 h and then incubated with the anti-STAT3 Y705 (Abcam, ab76315) and anti-H3K27Ac antibodies (Abcam, ab4729) for 1.5 h at room temperature. After the cells were washed and incubated with the secondary antibody, they were counterstained with DAPI and mounted for observation. Cell images were obtained with an Olympus FV1000 confocal microscope.

### Immunoprecipitation assay

The immunoprecipitation assay was performed using the Immunoprecipitation Protein G Dynabeads^®^ kit (Invitrogen, 10007D) according to the manufacturer’s protocol.

### Luciferase assay

For generation of luciferase reporters for the promoter assay, three luciferase reporter constructs that contained inserted sequences from – 500 to +500 bp relative to the TSSs of CAV2, TGFB2 and TGFBR1 were purchased from Shanghai GenePharma Co., Ltd. The luciferase activity was assayed using the Dual-Luciferase Reporter (DLR™) System (Promega, E1960) according to the manufacturer’s protocol. In the DLR™ Assay, the firefly and Renilla luciferase activities are measured sequentially from a single sample, and the ratio of firefly to Renilla luciferase is used as an internal control for the transfection efficiency.

### ChIP assay

The ChIP assay was conducted according to Dahl’s protocol [60]. Briefly, the cells were fixed with 1% formaldehyde and sonicated to shear the DNA. After centrifugation, the supernatants were incubated with H3K4Me3 (Abcam, ab8580), H3K27Me3 (Abcam, ab6002), H3K27Ac (Abcam, ab4729), H3K27Cro (Jingjie PTM Biolab, PTM-501), STAT3 Y705 (Abcam, ab76315) or RNAP II antibodies (Abcam, ab5131). Chromatin DNA was purified with protein G Dynabeads (Invitrogen, 10004D) and subjected to real-time PCR. The region-specific primers used are listed in Table S1.

### TMT-labelling and quantitative proteomics analysis

After the siNEAT1v2 and siCTRL cells were harvested, proteins were isolated and digested with trypsin. Then, the digested peptides were labelled with TMT using the TMT kit/iTRAQ kit according to the manufacturer’s protocol. The lysine crotonylated and acetylated peptides were enriched using pre-washed antibody beads (PTM Biolabs, Hangzhou) and subjected to an NSI source, followed by tandem mass spectrometry (MS/MS) in the Q Exactive™ (Thermo) coupled online to the UPLC. The resulting MS/MS data were processed using the Maxquant search engine (v.1.5.2.8).

### Flow cytometry

To investigate the functions of NEAT1 in the uptake and clearance of Aβ, HiLyte™ Fluor 488-labelled β-amyloid (1–42) (ANASPEC, AS-60479-01) was added to the siNEAT1v2 and siCTRL cell lines for the indicated times. Then, flow cytometry was performed on the BD AccuriC6 (BD Biosciences), and the data were analysed using FlowJo.

### Cell Counting Kit-8 assay

To determine the cell viability and proliferative capacity, U251 cells incubated with 80 µm crotonyl-CoA were plated into 96-well plates at a density of 2 × 10^4^ cells/well. After growth for 0, 12 and 24 h, 10 μl of CCK-8 (DoJinDo, CK04) was added to each well. The plates were incubated for an additional 2 h, and the absorbance was detected at a wavelength of 450 nm.

### Acetyl-coenzyme A assay

To determine whether NEAT1 regulated the generation of acetyl-CoA, U251 cells were transiently transfected with NEAT1 or negative control siRNAs for the indicated times. Then, the cell lysates were harvested and used to measure the acetyl-CoA level with the acetyl-coenzyme A assay kit (Sigma-Aldrich, MAK039) according to the manufacturer’s protocol.

### Statistics

The data are expressed as the mean ± SD from experiments repeated three times. *p < 0.001. Comparisons between two groups were performed using a two-sample *t* test. For three or more groups, standard one-way analysis of variance (ANOVA) with Bonferroni’s test was conducted. A two-tailed probability value < 0.05 was considered statistically significant.

### Electronic supplementary material

Below is the link to the electronic supplementary material.

Supplementary material 1 (DOCX 29010 kb)
