# Supplementary material for: NEAT1 regulates neuroglial cell mediating Aβ clearance via the epigenetic regulation of endocytosis-related genes expression
Source: Cell Mol Life Sci. 2019 Apr 20;76(15):3005–18. doi: 10.1007/s00018-019-03074-9 (PMC6647258; doi:10.1007/s00018-019-03074-9)
Supplement: Supplementary file 1 — Supplementary material 1 (DOCX 29010 kb) [file 18_2019_3074_MOESM1_ESM.docx]

**NEAT1 regulates neuroglial cell mediating Aβ clearance via the epigenetic regulation of endocytosis-related genes expression**

**Ziqiang Wang^1,2,3,†^, Yiwan Zhao^1,2,†^, Naihan Xu^2,4,5†^, Shikuan Zhang^1,2^ , Songmao Wang^2,4^, Yunhao Mao^2,4,5^, Yuanchang Zhu^1,2,4^, Bing Li^1,2,4^, Yuyang Jiang^4^, Ying Tan^2,4,5^, Weidong Xie^2,4,5^, Burton B Yang^6*^, Yaou Zhang^2,4,5,*^**

1 School of Life Sciences, Tsinghua University, Beijing 100084, China;

2 Key Lab in Healthy Science and Technology, Division of Life Science, Graduate School at Shenzhen, Tsinghua University, Shenzhen 518055, China;

3 Sun Yat-sen University Cancer Center, State Key Laboratory of Oncology in South China, Collaborative Innovation Center of Cancer Medicine, Guangzhou 510060, China;

4 State Key Laboratory of Chemical Oncogenomics, Graduate School at Shenzhen, Tsinghua University, Shenzhen 518055, China;

5 Open FIESTA Center, Tsinghua University, Shenzhen 518055, China;

6 Sunnybrook Research Institute and Department of Laboratory Medicine and Pathobiology, University of Toronto, Toronto, Canada.

†These authors contributed equally to this work.

*To whom correspondence should be addressed to Yaou Zhang. Email: zhangyo@sz.tsinghua.edu.cn, Correspondence may also be addressed to Burton B Yang. Email: byang@sri.utoronto.ca.

*Corresponding author, Key Lab in Healthy Science and Technology, Division of Life Science, Graduate School at Shenzhen, Tsinghua University, Shenzhen, P.R. China, phone: (86) 755-26036884, fax: (86) 755-26036884

E-mail: zhangyo@sz.tsinghua.edu.cn

The authors have declared that no conflict of interest exists.

**Supplementary material**

**
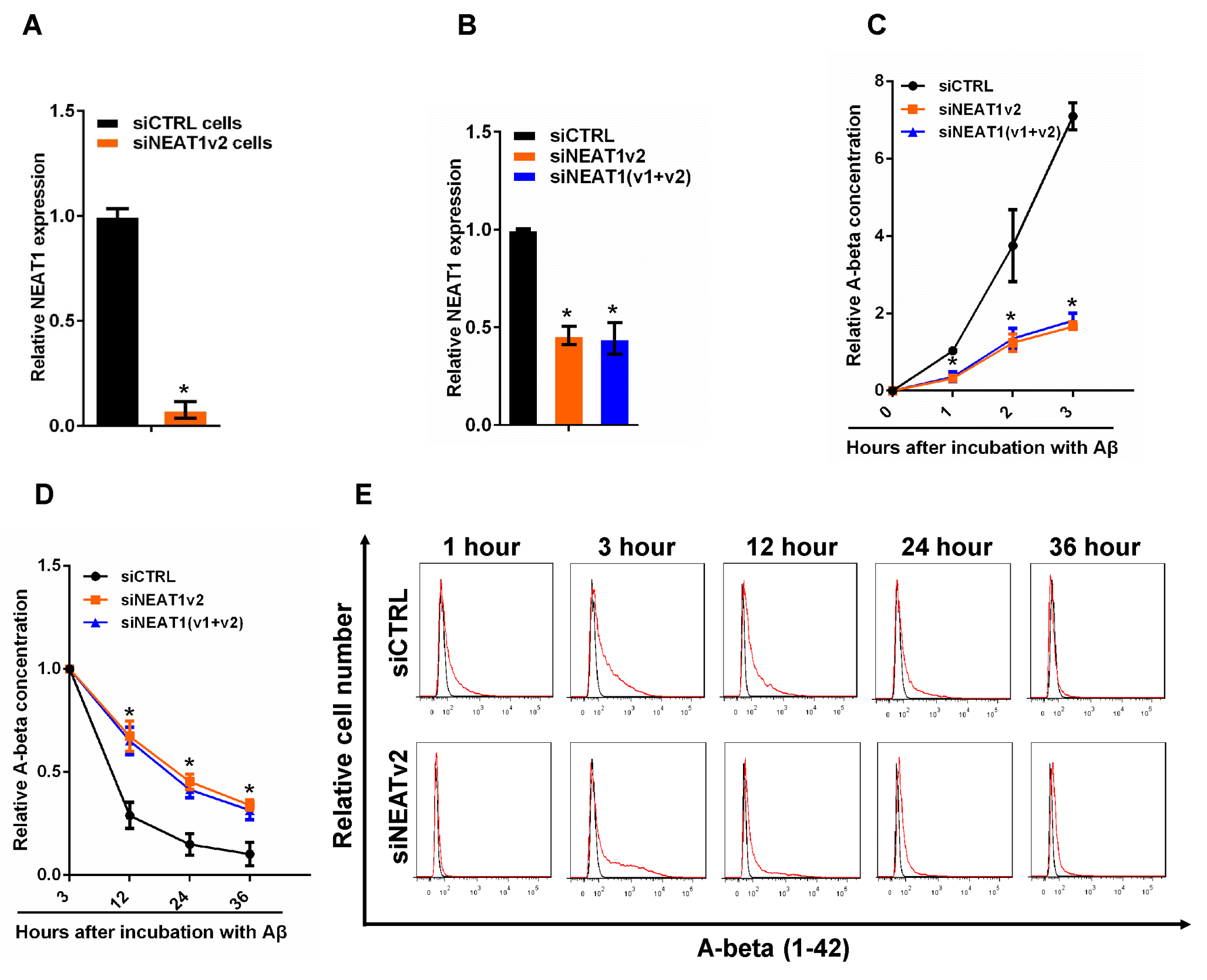
**

**Figure S1. NEAT1 regulates Aβ uptake and degradation.** (A). The relative expression of NEAT1 was analysed by qPCR in U251 cells stably expressing NEAT1-target shRNAs (siNEAT1 cells) or negative control shRNAs (siCTRL cells). The data points represent mean values determined from three independent experiments. The data are presented as the mean ± SD. (B). The relative expression of NEAT1 was analysed by qPCR in U251 cells transfected with siNEAT1v2, siNEAT1(v1+v2) or negative control siRNAs. The data points represent mean values determined from three independent experiments. The data are presented as the mean ± SD. (C, D). After Aβ (1-42) was added to U251 cells transfected with siNEAT1v2, siNEAT1(v1+v2) or negative control siRNAs for the indicated time points, the relative concentrations of Aβ (1-42) were analysed by ELISA in three independent experiments. The data points represent mean values determined from three independent experiments. The data are represented as the mean ± SD. (E). After Aβ (1-42) was added to siNEAT1 cells and siCTRL cells for the indicated time points, the concentrations of Aβ (1-42) were analysed by Flow Cytometry. The area under the black line indicate the basal signals obtained from cells, whereas the area under the red line indicate signals obtained from these cells that was added with HiLyte Fluor™ 488-labeled Aβ (1-42) for the indicated time points.*p < 0.001.


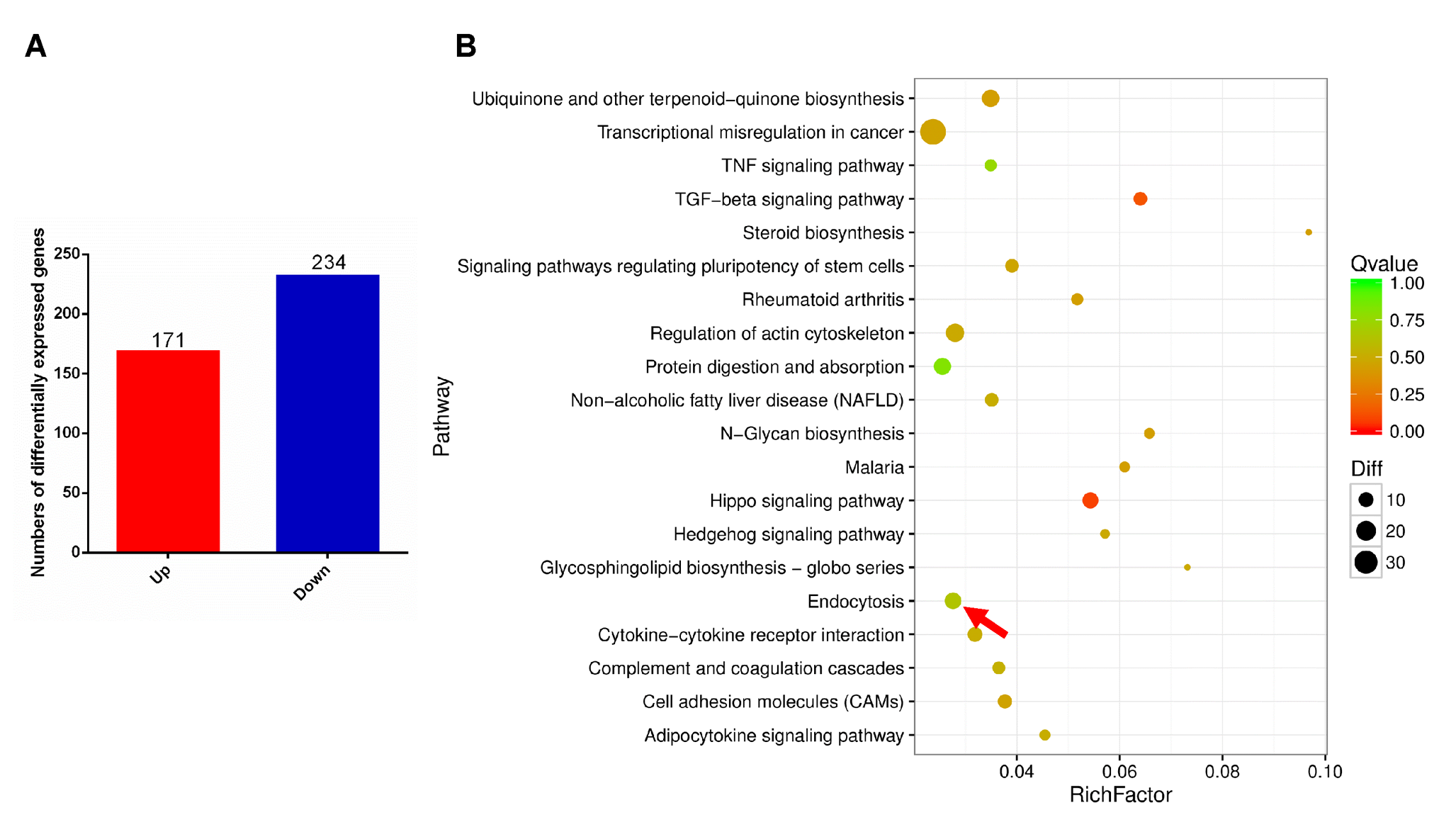


**Figure S2.** **The pathway regulated by NEAT1.** (A and B). Numbers of differentially expressed genes were analysed in RNA-seq results (A) and KEGG pathway analysis was conducted (B). The cut off for up/down regulation of genes is the fold change ≥2 and Q-value ≤0.01. Rich factor refers to the ratio of the number of differentially enriched genes to the number of annotated genes in the pathway. The larger the Rich factor, the greater the degree of enrichment. Qvalue is corrected Pvalue by multiple hypothesis test. The value range of Qvalue is from 0 to 1. The nearer to zero Qvalue is, the more significant the enrichment is. The size of dots indicates the number of differentially expressed genes.


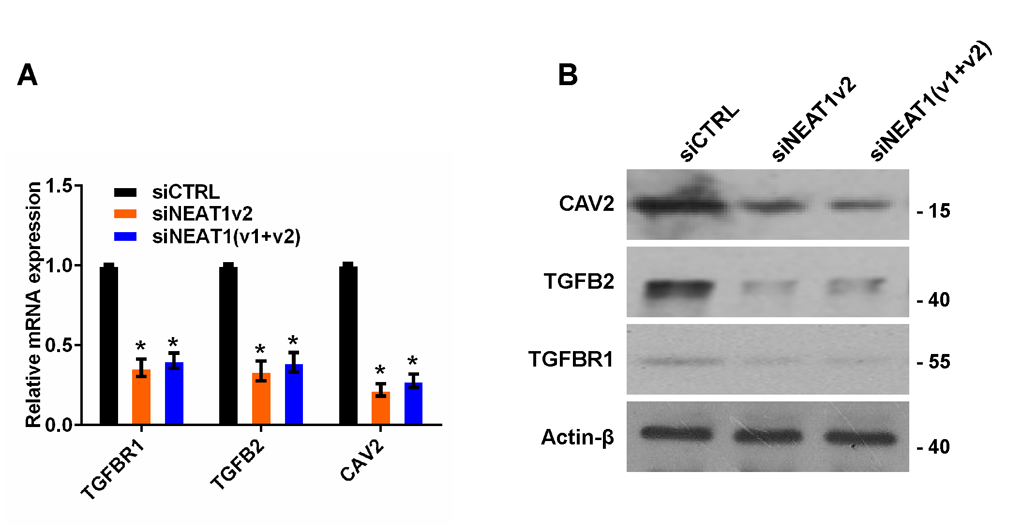


**Figure S3. NEAT1 regulates endocytosis-related gene expression.** (A). Relative *CAV2*, *TGFB2* and *TGFBR1* mRNA levels in U251 cells transfected with siNEAT1v2, siNEAT1(v1+v2) or negative control siRNAs were analysed by qRT-PCR in three independent experiments. The data are represented as the mean ± SD. (B). CAV2, TGFB2, TGFBR1 and Actin-β protein levels in U251 cells transfected with siNEAT1v2, siNEAT1(v1+v2) or negative control siRNAs were measured by western blotting. *p < 0.001.

**
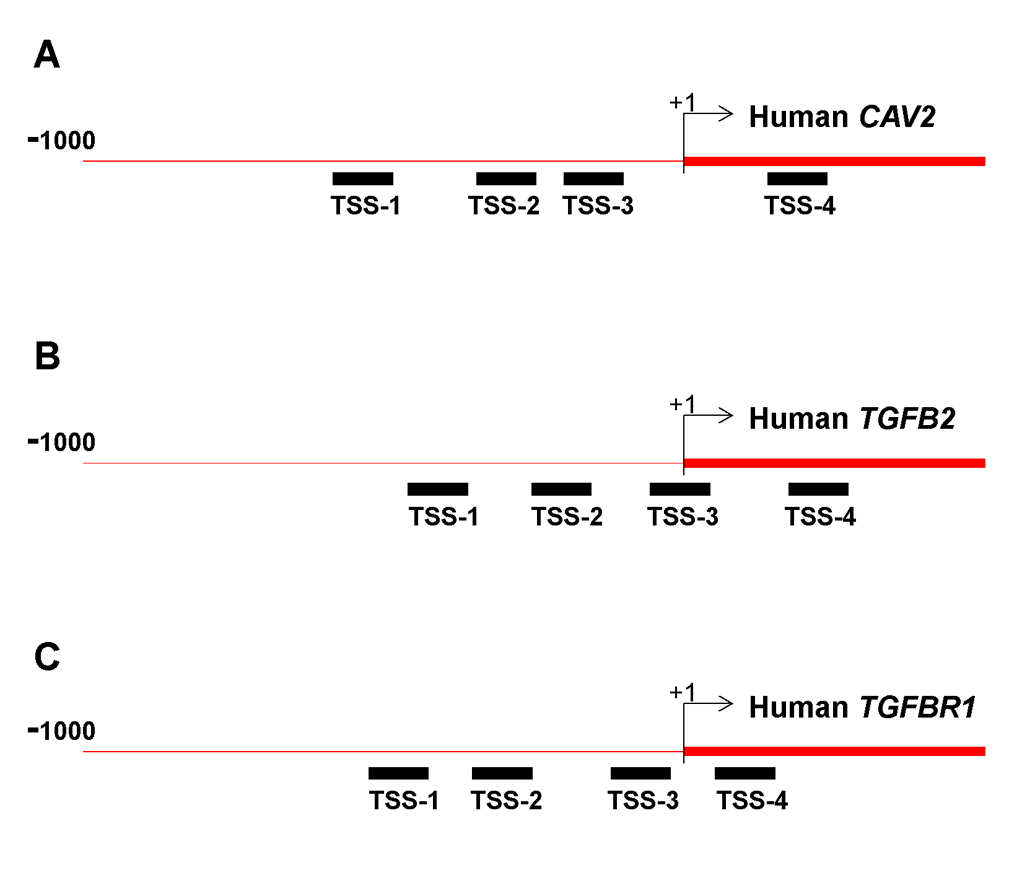
**

**Figure S4**. **The primer pairs designed to recognize the TSSs regions of genes.** Schematic diagram shows the gene structure of *CAV2*, *TGFB2* and *TGFBR1*, in which the black boxes represent the primer-amplified regions.

**
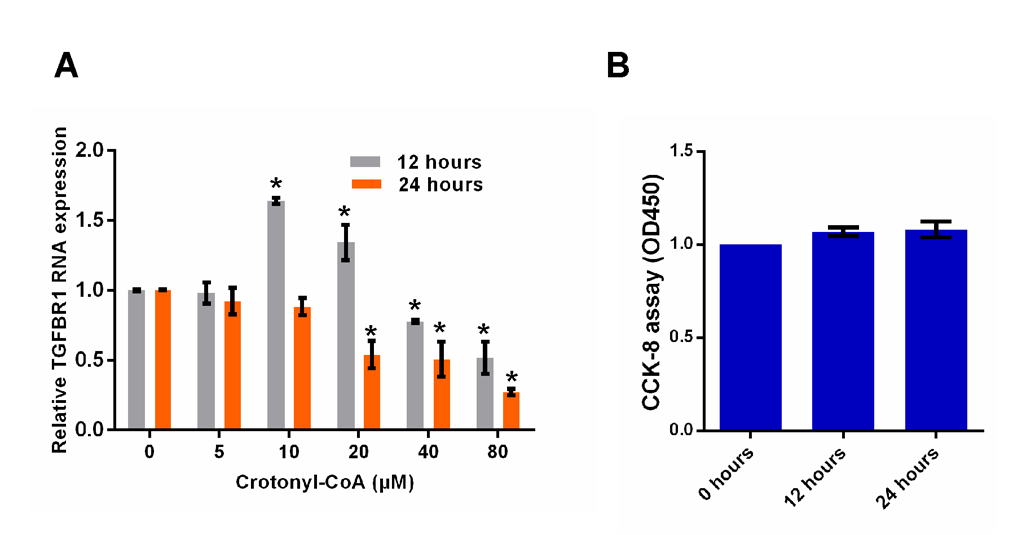
**

**Figure S5.** **Crotonyl-CoA regulates genes expression.** (A). The relative expression of TGFBR1 was analysed by qRT-PCR in U251 cells incubated with indicated concentration of crotonyl-CoA for 12 hours and 24 hours. The data points represent mean values determined from three independent experiments. The data are presented as the mean ± SD. (B). CCK8 assay was performed to determine the cell viability and proliferative ability of U251 cells incubated with 80 µm crotonyl-CoA for 12 hours or 24 hours. The data points represent mean values determined from three independent experiments. The data are presented as the mean ± SD. *p < 0.001.


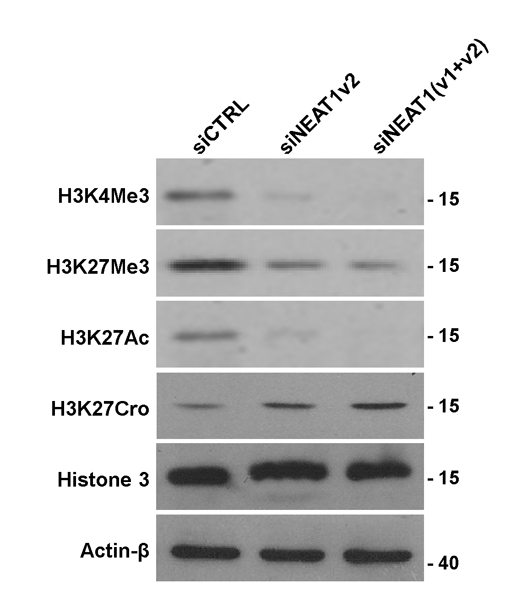


**Figure S6. NEAT1 regulates histone modification.** Levels of H3K4 H3K4Me3, H3K27Me3, H3K27Ac, H3K27Cro, Histone 3 and Actin-βin in U251 cells transfected with siNEAT1v2, siNEAT1(v1+v2) or negative control siRNAs was measured by western blotting.

**
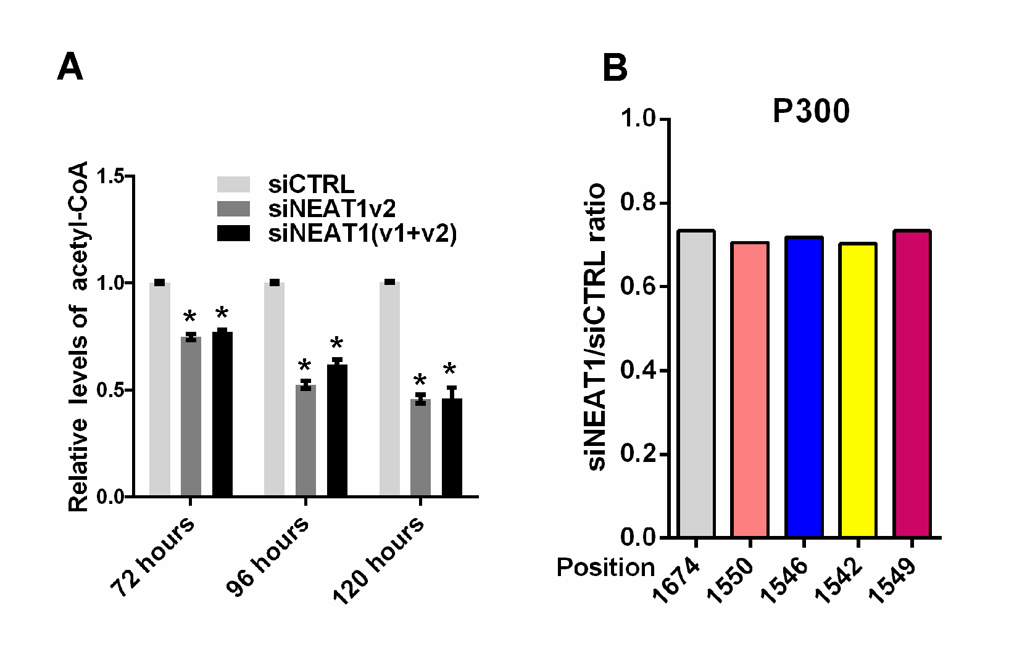
**

**Figure S7.** **NEAT1 influences acetyl-CoA generation and the autoacetylation of P300.** (A). Acetyl-Coenzyme A Assay was performed to determine the acetyl-CoA of U251 cells transfected with NEAT1 siRNAs (siNEAT1) or negative control shRNAs (siCTRL) for 72 hours, 96 hours and 120 hours. The data points represent mean values determined from three independent experiments. The data are presented as the mean ± SD. (B). After the modification-specific (lysine acetylation and crotonylation) proteomics analysis was performed in siNEAT1v2 cells and siCTRL cells, the differentially modified modification sites of P300 were analysed. *p < 0.001.


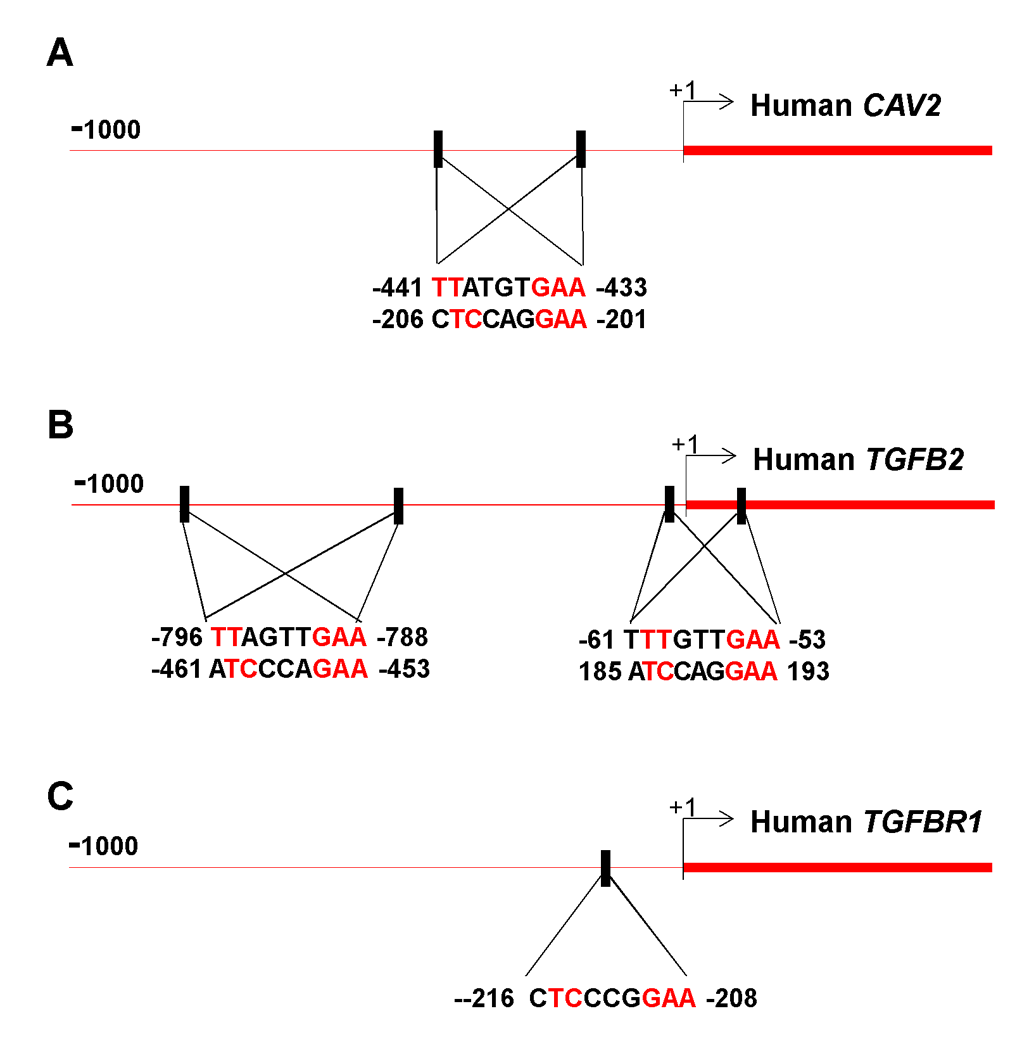


**Figure S8. The association of transcriptional factor STAT3 with targeted genes.** (A-C). Schematic representation of the STAT3-binding site in the human *CAV2* (A), *TGFB2* (B) or *TGFBR1* (C) gene. The black box shows the potential binding site, and the red characters indicate matching sequences.

**Table S1. Sequences of primers and siRNAs used in this study.**

**Name sense sequence antisense sequence**

| \| \| **SiRNAs** \| \| \|  \| \| --- \| --- \| --- \| --- \| \| NEAT1v2 siRNA \| CAAACUCUGUACCCAUUAA \| UUAAUGGGUACAGAGUUUG \|  \| \| NEAT(v1+v2) siRNA \| GUGAGAAGUUGCUUAGAAA \| UUUCUAAGCAACUUCUCAC \|  \| \| CAV2 siRNAs \| AGACCUGCCUAAUGGUUCU \| AGAACCAUUAGGCAGGUCU \|  \| \| TGFB2 siRNAs \| CCAAGUCAGACGUUAACAA \| UUGUUAACGUCUGACUUGG \|  \| \| TGFBR1 siRNAs \| GAAUGGAACUUGCUGUAUU \| AAUACAGCAAGUUCCAUUC \|  \| \| Negative control siRNA \| UUCUCCGAACGUGUCACGU \| ACGUGACACGUUCGGAGAA \|  \| \| **Primers pairs for Real Time PCR** \| \| \| \| \| NEAT1 \| ACATTGTACACAGCGAGGCA \| CATTTGCCTTTGGGGTCAGC \|  \| \| CAV2 \| ACAGCTCTTCATGGACGACG \| GGCATTAAAATCCTTGAGATGCGA \|  \| \| TGFB2 \| GCGACGAAGAGTACTACGCC \| GGCATCAAGGTACCCACAGA \|  \| \| TGFBR1 \| CCTCGAGATAGGCCGTTTGT \| AGGTGATGACTTTACAGTAGTTGGA \|  \| \| Actin-beta \| TGACGTGGACATCCGCAAAG \| CTGGAAGGTGGACAGCGAGG \|  \| \| **Primer pairs for ChIP** \| \| \|  \| \| CAV2 TSS-1 \| AGCTCTTCATGGACGACGAC \| TTGAGATGCGAGTTGAGCCG \|  \| \| CAV2 TSS-2 \| CTCCAGGAAGGCCGTTGTCT \| GGCAGCTGGGACGAGGA \|  \| \| CAV2 TSS-3 \| CCCAGAGGCTCAATACCAGC \| AGGGAACCTTGTGGTTAGGC \|  \| \| CAV2 TSS-4 \| CCTCTCACGACAGCAGCAA \| TCAGGGGTGTCCGTGAGAAT \|  \| \| TGFB2 TSS-1 \| CTGCAGACAGGAGGAGACAG \| CCACCCTTTCCCAACCTCTG \|  \| \| TGFB2 TSS-2 \| GACGGTCTAGGGAGTCATCC \| TGGAATTGCTCGCTTAGGGT \|  \| \| TGFB2 TSS-3 \| GCAGACACGTGGTTCAGAGA \| TCCCTAGACCGTCAGGCTAA \|  \| \| TGFB2 TSS-4 \| AAATCACTGTTGTCAGGGCG \| TTAGACCACGAGCTCTCCCC \|  \| \| TGFBR1 TSS-1 \| TGGGGATCCTCCCTTTCCAA \| CACCCAGTTCCAAACCCAGA \|  \| \| TGFBR1 TSS-2 \| GTGGAGCGTCTCGCAGTAAA \| GGGTTCCCCCTTCTTAGCAC \|  \| \| TGFBR1 TSS-3 \| AAGGAAGCCTTCACAGGTGG \| ACAGCCAAGGAGGCTGTTAC \|  \| \| TGFBR1 TSS-4 \| GTGTAGAGCAAGTTGCAGGC \| GCGTGGATATTGGAGCTGGG \|  \| \|  \| \| --- \| --- \| --- \| --- \| --- \| --- \| --- \| --- \| --- \| --- \| --- \| --- \| --- \| --- \| --- \| --- \| --- \| --- \| --- \| --- \| --- \| --- \| --- \| --- \| --- \| --- \| --- \| --- \| --- \| --- \| --- \| --- \| --- \| --- \| --- \| --- \| --- \| --- \| --- \| --- \| --- \| --- \| --- \| --- \| --- \| --- \| --- \| --- \| --- \| --- \| --- \| --- \| --- \| --- \| --- \| --- \| --- \| --- \| --- \| --- \| --- \| --- \| --- \| --- \| --- \| --- \| --- \| --- \| --- \| --- \| --- \| --- \| --- \| --- \| --- \| --- \| --- \| --- \| --- \| --- \| --- \| --- \| --- \| --- \| --- \| --- \| --- \| --- \| --- \| --- \| --- \| --- \| --- \| --- \| --- \| --- \| --- \| --- \| --- \| --- \| --- \| --- \| --- \| --- \| --- \| --- \| |  |  |
| --- | --- | --- | --- | --- | --- | --- | --- | --- | --- | --- | --- | --- | --- | --- | --- | --- | --- | --- | --- | --- | --- | --- | --- | --- | --- | --- | --- | --- | --- | --- | --- | --- | --- | --- | --- | --- | --- | --- | --- | --- | --- | --- | --- | --- | --- | --- | --- | --- | --- | --- | --- | --- | --- | --- | --- | --- | --- | --- | --- | --- | --- | --- | --- | --- | --- | --- | --- | --- | --- | --- | --- | --- | --- | --- | --- | --- | --- | --- | --- | --- | --- | --- | --- | --- | --- | --- | --- | --- | --- | --- | --- | --- | --- | --- | --- | --- | --- | --- | --- | --- | --- | --- | --- | --- | --- | --- | --- | --- |
